# Comment on “Effect of Exercise Intervention on Flow-Mediated Dilation in Overweight and Obese Adults: Meta-Analysis”

**DOI:** 10.1155/2018/5082903

**Published:** 2018-06-05

**Authors:** Mohammad Alwardat

**Affiliations:** School of Neuroscience, Faculty of Medicine and Surgery, University of Rome “Tor Vergata”, Rome, Italy

Son et al. [1] recently conducted systematic review and meta-analysis that evaluated the effects of exercise intervention on flow-mediated dilation in overweight and obese adults. Evaluating the effectiveness of physical activity and exercise can enhance the awareness of these factors for flow-mediated dilation for both overweight and obese adults. However, we have some comments with respect to the procedures and results of this study.

First, the authors assessed the quality of the included randomized clinical trials using the PEDro scale. A cutoff score of 6 was used to assess the level of evidence. In a randomized clinical trial, a score of 6 or higher was considered to be of moderate to high quality of evidence, whereas a score of less than 6 was considered to be of low quality of evidence. A score of 6 on PEDro is not sufficient to consider moderate to high quality of evidence especially for the randomized clinical trials that compared exercise interventions with usual physical exercise or no interventions. Those articles have better possibility of matching blinding patients and therapists. A metaepidemiological study published in 2014 found that PEDro and Cochrane approaches to identifying RCTs of adequate quality lead to different sets of trials and different combined treatment estimates from meta-analyses of these trials [2]. We believe that adapting the Grading of Recommendations Assessment and Development and Evaluation GRADE approach is highly recommended and efficient.

Second, the inclusion criteria are ambiguous. The authors do not sufficiently follow the PICO format (P: participants, I: intervention, C: comparison, O: outcomes). The authors state the inclusion criteria as follows: “Study included the value of relative FMD; included exercise intervention at least 7 days; considered only overweight and/or obese adults; is written in English language and published in peer-reviewed journals through June 2016”. Additionally, the authors state that the review aims to “evaluate the relationship between exercise training and EF in overweight and obese adults”. Surprisingly, the review did not clearly report the other components of inclusion criteria like comparison and outcome measures. The included articles were RCTs that compared the effect of different types of exercise (such as aerobic, resistance, and combined exercise) in overweight and obese adults. Running a systematic review without full knowledge about the inclusion criteria can lead to problems with assessing the validity, applicability, and comprehensiveness of the systematic review [3].

Finally, the systematic review is different from other types of literature reviews. It must provide an explicit, reproducible methodology and include a systematic search that attempts to identify all studies that would meet the eligibility criteria [4]. This unique construction requires the Methods section of a systematic review to be evaluated much like a quantitative research study. However, this review has also several troubling flaws in the methods. The authors reported using PubMed; there was also the opportunity to use Medical Subject Headings (MeSH) in the search. Using subject headings in addition to keywords is a key point of searching for studies according to Cochrane Handbook for Systematic Reviews of Interventions [4].
